# Immunomodulation through Nutrition Should Be a Key Trend in Type 2 Diabetes Treatment

**DOI:** 10.3390/ijms25073769

**Published:** 2024-03-28

**Authors:** Katarzyna Napiórkowska-Baran, Paweł Treichel, Marta Czarnowska, Magdalena Drozd, Kinga Koperska, Agata Węglarz, Oskar Schmidt, Samira Darwish, Bartłomiej Szymczak, Zbigniew Bartuzi

**Affiliations:** 1 Department of Allergology, Clinical Immunology and Internal Diseases, Collegium Medicum Bydgoszcz, Nicolaus Copernicus University Toruń, 85-067 Bydgoszcz, Poland; zbartuzi@cm.umk.pl; 2 Student Research Club of Clinical Immunology, Department of Allergology, Clinical Immunology and Internal Diseases, Collegium Medicum Bydgoszcz, Nicolaus Copernicus University Toruń, 85-067 Bydgoszcz, Poland; treichel.pawel@gmail.com (P.T.); czarnowska.marta@onet.pl (M.C.); magdalena.drozd515@wp.pl (M.D.); kingakoperska@gmail.com (K.K.); agataklaraweglarz@gmail.com (A.W.); okischmidt@wp.pl (O.S.); samiradarwish00@gmail.com (S.D.); bartlomiej.szymczak1@gmail.com (B.S.)

**Keywords:** type 2 diabetes, immunomodulation, immune system, nutrients, macroelements, microelements, vitamins, omega-3 acids, coenzyme Q10, alpha-lipoic acid

## Abstract

An organism’s ability to function properly depends not solely on its diet but also on the intake of nutrients and non-nutritive bioactive compounds that exert immunomodulatory effects. This principle applies both to healthy individuals and, in particular, to those with concomitant chronic conditions, such as type 2 diabetes. However, the current food industry and the widespread use of highly processed foods often lead to nutritional deficiencies. Numerous studies have confirmed the occurrence of immune system dysfunction in patients with type 2 diabetes. This article elucidates the impact of specific nutrients on the immune system function, which maintains homeostasis of the organism, with a particular emphasis on type 2 diabetes. The role of macronutrients, micronutrients, vitamins, and selected substances, such as omega-3 fatty acids, coenzyme Q10, and alpha-lipoic acid, was taken into consideration, which outlined the minimum range of tests that ought to be performed on patients in order to either directly or indirectly determine the severity of malnutrition in this group of patients.

## 1. Introduction

Diabetes is a disease that affects a growing number of people worldwide. It is one of the leading causes of disability and mortality regardless of origin, gender, or age. In 2021, an estimated 536.6 million people aged 20–79 suffered from diabetes globally, of which approximately 96% were type 2 diabetes cases, predominantly among the elderly. The burgeoning problem of diabetes was associated with the co-occurrence of a high BMI in more than 50% of cases. Unfortunately, recent analyses suggest that the problem will continue to grow, and by 2045, more than 783.2 million people aged 20–79 will have developed diabetes. It is noteworthy that nearly one-third of deaths from diabetes and its complications occur in people under the age of 60 [1,2].

Diabetes mellitus is not just a health concern but also an economic matter. The estimated cost of diabetes-related economic burdens among people aged 20–79 has increased by as much as 366% between 2007 and 2021, reaching USD 966 billion, and it is projected to reach USD one trillion by 2030 [2].

Complications of diabetes pose a major challenge in treatment and may be divided into two categories: macrovascular and microvascular. Macrovascular complications include cardiovascular diseases such as coronary artery disease, peripheral vascular disease, and cerebrovascular disease. Microvascular complications of diabetes include diabetic retinopathy, diabetic nephropathy, and diabetic neuropathy. Approximately 50% of patients with type 2 diabetes experience macrovascular complications, while microvascular complications occur in 27% of patients. These complications have a significant impact on patient mortality and quality of life [3,4].

Treating diabetes requires a holistic approach, which includes physical activity, appropriate diet, pharmacological treatment, education, and continuous motivation of the patient. In some cases, the assistance of a psychologist and/or psychiatrist may also be expedient.

According to the still valid concept of Marc Lalonde, modifiable factors have the greatest impact on human health, approximately 70%, and lifestyle as much as 50% [5]. A proper diet is undeniably crucial in managing diabetes. However, the food industry’s current practices are not conducive to proper nutrition due to the high extent of food processing, the ubiquitous use of food additives, and, above all, the conditions under which plants are grown. Many studies confirm a systematic decrease in the concentrations of microelements, macroelements, and vitamins in plant-based products, which should be the foundation of a well-balanced diet [6,7,8]. Consequently, clinicians treat malnourished patients of normal or excessive weight, as their bodies lack non-nutritive bioactive compounds. 

The immune system is the most important system in the human body, and it determines its efficient functioning in every aspect. Its prominence also extends to the impact on the development and course of chronic diseases, including diabetes. Nevertheless, in order for the immune system to operate efficiently, it requires not only protein (essential, among others, for the synthesis of key enzymes) but most importantly, non-nutritive bioactive compounds [9,10]. 

Immunomodulation through nutrition, alternatively referred to as immunonutrition, is a field of science that relates aspects of nutrition, the functioning of the immune system, and the impact of inflammation and pathological processes in the body. Its beneficial effect has been demonstrated in many conditions, such as gastrointestinal cancer or Crohn’s disease [11,12]. The use of immunomodulation through appropriate nutrition should also be a priority in such an important disease as diabetes.

## 2. Methods

### 2.1. Search Strategy and Sample Period

A systematic literature review adhering to the guidelines outlined in the Preferred Reporting Items for Systematic Reviews and Meta-Analyses (PRISMA) recommendations was undertaken. Electronic databases, including PubMed and Google Scholar, were comprehensively searched from their respective inception dates from 15 December 2023 to 5 February 2024. Key search phrases included “type 2 diabetes treatment” (step 1), “novel therapeutic options in the treatment of type 2 diabetes”, “coenzyme Q10 in type 2 diabetes”, “macroelements in type 2 diabetes”, “microelements in type 2 diabetes”, “omega-3 fatty acids in type 2 diabetes”, and “vitamins in type 2 diabetes” (step 2). In step 3, the phrase “type 2 diabetes” + a specific parameter was searched, i.e., individual drugs, microelements, macroelements, vitamins, and other compounds. The reference lists of all the included articles were thoroughly screened to find other relevant articles. After eliminating duplicate entries, reviewers independently assessed all titles and abstracts, followed by a full-text evaluation of eligible articles based on predefined inclusion criteria. Any discrepancies among reviewers involved in the literature search were resolved through discussion with all authors to achieve consensus.

### 2.2. Selection Criteria

**Figure 1:**
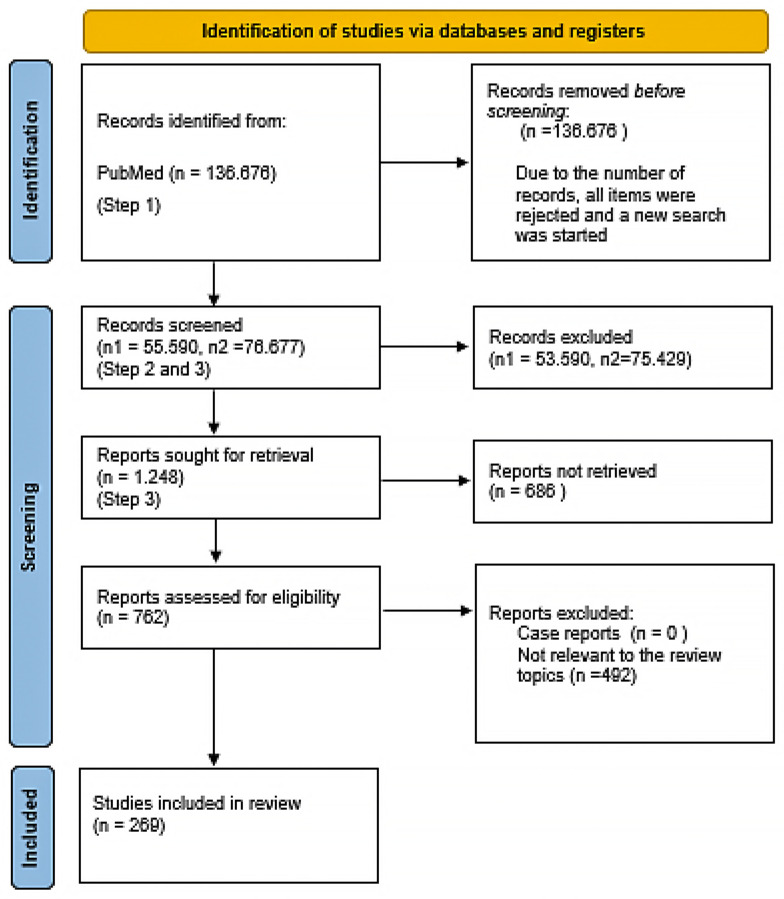
PRISMA flowchart.

Studies were considered eligible if they met the following inclusion criteria: (i) relevance to the topic and (ii) presentation of clinical evidence supporting the research hypothesis. Exclusion criteria included (i) case reports and (ii) studies lacking accessibility to their underlying data. Identification of publications was rigorously performed during the screening process. Figure 1 shows the flowchart according to the PRISMA statement.

## 3. Pathogenesis of Diabetes with Particular Emphasis on the Role of the Immune System

Obesity is a major risk factor for insulin resistance and, consequently, type 2 diabetes. Insulin resistance is caused, among others, by tumor necrosis factor alpha (TNF-α), the increased production of which is observed in people with excessive adipose tissue. Berbudi et al. observed that, in addition to increased levels of TNF-α, obese patients also have increased levels of C-reactive protein and plasminogen activator inhibitor in the blood. Reactive oxygen species, free fatty acids, and the aforementioned inflammatory cytokines activate Iҡβα kinase (IKKβ) and c-Jun N-terminal kinase I (JNK1), resulting in the inhibition of the insulin receptor substrate (IRS-1). In addition to inhibiting IRS-1, those kinases sway the activation of the transcription of inflammatory genes, which promote insulin resistance. IRS-1 is also inhibited by STAT tyrosine phosphorylation, which is induced by JAK kinase. As a consequence of both mechanisms, GLUT-4 translocation to cell membranes is impaired, leading to hyperglycemia [13].

Interleukins are inflammatory cytokines responsible for, among other things, defense against pathogens. Mooradian et al. observed that isolated monocytes in people with diabetes, regardless of the type, secreted less interleukin 1β (Il-1β) after stimulation with liposaccharides (LPS) [14]. Moreover, in other studies, the authors demonstrated that hyperglycemia causes a decrease in interleukin 2, 6, and 10 serum concentrations [15,16]. Of particular relevance in this combination is IL-6, which is responsible for the adaptive induction of antibody production and the development of effector T lymphocytes [13].

Kumar et al. proved that hyperglycemic mice have impaired infiltration of CD45+ leukocytes and CD8+ T lymphocytes. Additionally, a correlation of this phenomenon with a weaker expression of adhesion molecules, namely E-selectin and intracellular adhesion molecule (ICAM), is observed [17].

The matter concerning the expression of toll-like receptors (TLRs) in response to hyperglycemia remains unresolved. While some studies have reported a complete reduction in TLR expression, others have not observed significant change in patients with well-controlled hyperglycemia [18,19].

In obese patients, who constitute the largest group of diabetic patients, the level of resistin, a protein that stimulates the endothelium to store lipids, is higher. Increased resistin levels have a negative impact on the production of reactive oxygen species by neutrophils (ROS) [20]. Under hyperglycemic conditions, neutrophil ROS production is diminished, degranulation is impaired, phagocytosis is decreased, neutrophil extracellular matrix (NET) formation is weakened, and antibody opsonization is negatively affected [13].

The impairment of multitudinous immune mechanisms by hyperglycemia is also indicated by its effect on the complement system-reduced opsonization of the C4 fragment, and dysfunction of natural killer cells is observed [13].

Hyperglycemia is one of the conditions that can result in oxidative stress and the upregulation of pro-inflammatory factors. ROS are highly reactive molecules capable of independent existence, containing at least one oxygen atom and one or more unpaired electrons [21]. They are primarily generated in mitochondria as natural byproducts in all aerobic organisms. ROS can be also formed in other mechanisms, such as NADPH-oxidase, immune reactions, xanthine oxidase, arachidonic acid metabolism, and others. They are involved in the processes of intracellular signaling and cell activity [22]. In sundry metabolic disorders, such as diabetes, ROS can accumulate, and this excessive formation of free radicals leads to oxidative stress, causing damage at the molecular and cellular level [21]. Oxidative stress leads to impaired glucose uptake in muscle and fat cells and decreases insulin secretion from beta-cells. Hyperglycemia can also cause increased ROS production through the activation of the kinase C pathway via diacylglycerol, increased hexosamine pathway flux, increased advanced glycation end production, and increased flux in the polyol pathway [23]. Under hyperglycemic conditions, excessive production of ROS during glycolysis reactions is observed, and it has been proven to damage DNA and subsequently activate poly-ADP-ribose polymerase 1 (PARP1), which is a DNA repair enzyme. PARP1 inhibits glyceraldehyde-3-phosphate dehydrogenase (GA3PDG) activity, which leads to the accumulation of glyceraldehyde-3-phosphate (GA3P) and other glycolysis intermediates. The rise of GA3P content activates the mentioned pro-oxidant pathways. Moreover, GA3P accumulation can cause glucose autoxidation, which results in hydrogen peroxide formation that contributes to oxidative stress [22]. Mitochondrial destruction (caused by oxidative stress), along with the activation of the mentioned hexosamine pathway, nuclear factor-κB (Nf-κb), p38 mitogen-activated protein kinase (p38 MAPK), c-jun NH2 terminal kinase/stress-activated protein kinase (JNK/SAPK), or toll-like receptors (TLRs), leads to pancreatic β-cell dysfunction. Furthermore, oxidative stress is predisposed to decreased insulin production caused by the inhibition of the nuclear transcription factors Pdx-1 (insulin promoter factor 1) and MafA transcription factor, which impairs insulin secretion by opening the K_ATP_ channels and inhibiting calcium flow, while having also a pro-apoptotic effect on β-cells [24].

Diabetic patients develop chronic low-grade inflammation, which is reflected by high levels of cytokines, such as TNF-alpha and other pro-inflammatory markers, such as CRP. Hyperglycemia can directly stimulate the upregulation of cytokines, chemokines, and adhesion molecules, modulating various pathways that ultimately converge towards pathways containing transcription factor protein complexes controlling the transcription of DNA, namely NF-kB signaling [22]. The process of inflammation in diabetes is also affected by adipose tissue macrophages (ATMs). Those cells produce factors that act in a paracrine or systemic manner and interrupt insulin signaling in target cells. ATMs release exosomes that can exert local paracrine effects or may enter the circulation [25]. A recent study has shown that ATM-derived exosomes isolated from obese mice directly cause decreased insulin signaling in adipocytes, myocytes, or primary hepatocytes in vitro. These isolated exosomes contained high levels of miR-155, which suppresses PPAR-γ expression, thereby contributing to insulin resistance [25,26].

## 4. Novel Therapeutic Options in the Treatment of Type 2 Diabetes

The field of diabetes care is constantly developing, and new therapeutic options are emerging. Novel therapeutic possibilities can increase the quality of care and overall well-being of individuals with diabetes. Changes related to new research, technology, and treatments lead to updates in guidelines.

In 2023, the latest Standards of Care in Diabetes were released and within them, updated guidelines were established. The amendments concerned the incorporation of person-centered and inclusive language. Moreover, an additional language and some new definitions were introduced along with the benefits of using them in the telemedicine subsection. Recommendations regarding the use of statins and periodic monitoring of blood glucose levels in individuals at high risk of developing type 2 diabetes who have been prescribed statin therapy have been added. Additionally, the recommendation was introduced to the discussion part of pioglitazone use, which seems to reduce the risk of stroke or myocardial infarction in people with a history of stroke and evidence of insulin resistance and prediabetes. A dual GLP-1/glucose-dependent insulinotropic polypeptide (GIP) receptor agonist, commonly known as Tirzepatide, has been proposed as a glucose-lowering option that may cause weight loss. It was emphasized that either small or larger weight losses ought to be considered as treatment goals on a case-by-case basis [27].

Furthermore, the blood pressure treatment targets were changed, and it is currently recommended to achieve < 130/80 mmHg in individuals with diabetes. Pharmacological treatment should be considered in patients with diabetes and a blood pressure ≥ 130/80 mmHg. In people with diabetes aged 40–75 years with atherosclerosis risk factors, LDL cholesterol should be reduced to ≥50% of the baseline value, and a final LDL cholesterol level of <70 mg/dl should be achieved. On top of that, ezetimibe or a PCSK9 inhibitor should be used in such cases in order to achieve maximally tolerated statin therapy. In those with type 2 diabetes and diagnosed heart failure with either preserved or reduced ejection fraction, treatment with a sodium–glucose cotransporter 2 inhibitor is recommended [27].

Major changes were introduced in the immunization subsection to consider new indications and guidelines, especially regarding COVID-19 and pneumococcal pneumonia vaccinations, including age recommendations and bivalent boosters against COVID-19 [27].

Some attention has been given to nutrition, especially for diabetes in pregnancy. Nutrition counseling was endorsed to ameliorate the quality of diet, particularly the balance of macronutrients inclusive of nutrient-dense fruits, vegetables, legumes, whole grains, and healthy fats with omega n-3 fatty acids that are embodied in nuts, seeds, and fish in eating patterns [27].

### 4.1. Tirzepatide

Tirzepatide is a dual agonist of receptors of glucose-dependent insulinotropic polypeptide (GIP) and Glucagon-Like Peptide-1 (GLP-1) and, according to their activities, both play a role in controlling glucose blood levels [28]. 

GIP and GLP-1 are incretin hormones that are produced and released in the intestine in response to nutrient intake. Both stimulate beta cells of the pancreas to secrete insulin. GIP and GLP-1 are responsible for about 65% of postprandial insulin secretion [28,29]. The action of GIP and GLP-1 is related to the incretin effect. The phenomenon consists of enhancing the production and release of GIP and GLP-1 as a response to the increase in the concentration of glucose absorbed from the gut. Interestingly intravenous administration of glucose does not affect the secretion of GIP and GLP-1. Released GIP and GLP-1 stimulate pancreatic beta cells to secrete insulin and thus lower the blood concentration of glucose. The difference between insulin secretion in response to glucose absorbed from oral and intravenous administration of glucose is called the incretin effect. The intensity of this phenomenon depends on the quantity of glucose ingested. The insulinotropic activity of GIP and GLP-1 are dependent on plasma glucose concentrations, hence high levels of GIP and GLP-1 do not result in hypoglycemia. Dysfunction of the incretin effect is associated with impaired oral glucose tolerance and is referred to as either impaired glucose tolerance or diabetes [30,31]. 

Furthermore, both GIP and GLP-1 may affect other metabolic functions. GIP adversely affects gastric secretion activity. Additionally, GIP stimulates beta cells of the pancreas to secrete insulin. It has insulin-like activity on adipose tissue, where it inhibits lipolysis and promotes lipogenesis [27]. The activity of GLP-1 promotes insulin secretion and inhibits glucagon release [32,33]. What is more, GLP-1 reduces food carving and leads to a delay in gastric emptying, thereby preserving a sense of satiety and lowering body weight [31,34].

Tirzepatide is a new molecule that exhibits agonist activity towards GIP and GLP-1 receptors. It is the first so-called “twincretin” synthetic peptide, which is composed of 39 amino acids with a structure based on the native sequence of the GIP [35,36]. By stimulating GIP and GLP-1 receptors, Tirzepatide acts through the same mechanisms as native GIP and GLP-1 to control blood glucose and reduce body weight. It has been proved that Tirzepatide reduces the level of HbA1c and body weight in individuals with type 2 diabetes more effectively than other selective GLP-1 receptor agonists [31,37,38,39]. Tirzepatide has a five times lower affinity for the GLP-1 receptor than the native GLP-1, yet it binds to the GIP receptor with the same strength as native GIP [40,41]. 

Currently, Tirzepatide is indicated for treatment of type 2 diabetes mellitus (T2DM). According to the SURPASS trials, which assessed the safety and efficacy of Tirzepatide in people with T2DM, it emerged that Tirzepatide improved multiple cardiometabolic risk factors, such as a reduction in liver fat, new-onset macroalbuminuria, blood pressure, and lipid profile [39,42]. 

### 4.2. Thiazolidinediones

Pioglitazone belongs to the thiazolidinediones, and it is a peroxisome proliferator-activated receptor gamma (PPARγ) agonist. Pioglitazone use was proven to decrease the risk of myocardial infarctions and ischemic strokes. Currently, the use of this drug is indicated for individuals with T2DM and a stroke in medical history, evidence of insulin resistance, and prediabetes [43,44]. 

Peroxisome proliferator-activated receptors, widely known as PPARs, are a group of transcription factors that have a significant impact on glucose and lipid metabolism. Three isoforms have been discovered so far in mammals: PPARα (NR1C1), PPARβ/δ (NR1C2), and PPARγ (NR1C3). PPARs are mainly responsible for controlling genes involved in lipid metabolism, including transport, storage, lipogenesis, and fatty acid oxidation. PPARs are expressed in various types of cells throughout the body, such as pancreatic beta cells or cells of the immune system, and, therefore, PPARs play a role in regulating insulin secretion and T cell differentiation [45,46]. PPARγ is expressed in adipose tissue, intestines, liver, and kidneys, where it participates in the regulation of fat cell differentiation and lipid storage. Moreover, PPARγ has anti-inflammatory properties and has an influence on the differentiation of monocytes into macrophages, where it inhibits their conversion to the M2 phenotype [47,48]. It has been proven that polymorphisms in the PPAR β/δ and PPARγ promoter regions may result in a genetic predisposition to type 1 diabetes and can influence the severity of islet autoimmunity. Furthermore more, PPAR γ is correlated to the evolution of insulin resistance and type 2 diabetes. Therefore, PPARs have become an interesting target in the treatment of diabetes [49,50]. Thiazolidinediones through the activation of PPARγ receptors are able to directly decrease insulin resistance. The stimulation of PPARγ results in simplified differentiation of mesenchymal stem cells into adipocytes, increased lipogenesis in peripheral lipocytes, decreased hepatic and peripheral triglycerides, decreased visceral adipocyte activity, and increased adiponectin, which has anti-apoptotic effects in cardiomyocytes and pancreatic beta cells [51,52,53]. In addition, adiponectin has anti-inflammatory qualities and increases insulin sensitivity [54,55]. 

Pioglitazone, through the stimulation of PPARγ, promotes insulin sensitivity in skeletal and cardiac muscle and activates the insulin signal transduction system, which results in improved glucose transport and the enhancement glycogen synthesis and glucose oxidation. In addition, it increases glucose consumption by intensifying the function of mitochondria, thus reducing plasma-free fatty acid levels and promoting the reversal of lipotoxicity [56,57,58,59,60]. Pioglitazone reduces the risk of recurrence of major adverse cardiovascular events, myocardial infarction, stroke, and new-diagnosis dementia [60,61].

In vivo studies in rats and mice have proven that long-term treatment with rosiglitazone or troglitazone, both being PPARγ agonists, maintains beta-cell proliferation and prevents age-related loss of pancreatic mass in these animals. Additionally, troglitazone may prevent pancreatic abnormalities associated with age and increases in fasting insulin levels [45].

The number of diabetes cases is constantly rising and constitutes a significant demographic challenge. Therefore, new potential therapeutic targets for the treatment of diabetes are being explored in ongoing research, including GPCR 119, Vaspin, Metrnl, and Fetuin-A [62,63,64,65,66,67]. 

### 4.3. GPCR119 Receptor

GPR119 is a class-I G protein-coupled receptor found in skeletal and cardiac muscles, the liver, and pancreatic β-cells [63,68,69]. The activation of the GPR119 receptor leads to the activation of a cascade of Gα-stimulating proteins that induce adenylate cyclase activity. As a result, intracellular cyclic adenosine monophosphate (cAMP) increases, which may result in the release of GLP-1. The activation of GPR119 in pancreatic β-cell leads to glucose-stimulated insulin secretion similar to GLP-1 and GIP. Therefore, GPR119 plays a role in regulating glucose homeostasis and appetite [63,70,71]. Due to its dual activity and low risk of hypoglycemia, agonists of the GPR119 receptor are considered potential targets for the treatment of T2DM. So far, phase-2 clinical trials testing DS-8500 as a GPR119 agonist are ongoing [72].

The antidiabetic effect of DA-1241 as another GPR119 receptor agonist was tested in vitro and in vivo in mice. According to the results, it was described that although the administration of DA-1241 does not affect body weight gain and the quantity of food intake, fasting blood glucose level decreased along with an increase in the concentration of GLP-1. DA-1241 significantly improved solely the oral glucose tolerance test, with no changes in the intraperitoneal glucose tolerance test or the insulin tolerance test. DA-1241 caused a reduction in triglyceride content in the liver, which led to improvement in the fatty liver. The outcomes of the research suggested that DA-1241 has a substantial effect on glucose-dependent insulin release by the stimulation of GLP-1 secretion and contributes to reduced hepatic gluconeogenesis [73]. 

### 4.4. Vaspin 

Vaspin, also known as Serpin A12, is an adipokine that is a part of the serum and originally comes from fat cells. It has an insulin-sensitizing effect and contributes to reduced food intake. In studies conducted on rats, it was observed that the administration of Vaspin resulted in an improvement in insulin sensitivity along with increased glucose tolerance [62,74,75].

Vaspin has a significant effect on insulin modification and plays a role in modulating adipocyte differentiation and glucose homeostasis [76,77]. Moreover, Vaspin is an inhibitor of the kallikrein 7 (KLK7), which is responsible for the degradation of insulin, which, consequently, reduces its concentration. By influencing KLK7, Vaspin contributes to the enhancement of insulin signaling and extends the half-life of insulin, which leads to an increased insulin concentration and effects blood glucose levels [75]. Vaspin has the effect of reducing inflammatory adipokines; therefore, it may influence inflammatory processes. This action may contribute, to some extent, to improving insulin resistance [78].

### 4.5. Metrnl 

Metrnl is an adipokine that originally comes from the adipose tissue, particularly from the subcutaneous white fat, but may also be traced in the intestine and epithelium of the respiratory tract [79,80].

Metrnl contributes to the increase in lipid metabolism and diminishes inflammation caused by a high-fat diet. By acting on PPARγ receptors, it intensifies the remodeling of adipose tissue, which also improves insulin resistance and promotes the expression of GLUT4 receptors in skeletal muscle. Therefore, Metrnl influences insulin sensitivity and reduces inflammation [65,81,82]. In addition, decreased serum Merntl levels were reported in individuals with T2DM and with newly diagnosed T2DM [83,84,85,86]. It has been proven that Metrnl deficiency may dwindle HDL blood concentration and augment plasma levels of triglycerides. As a result, it has the additional function of controlling altered blood lipids [65,87].

### 4.6. Fetuin-A

Fetuin-A is a glycopeptide synthesized mainly in the liver. So far, many functions have been discovered [88,89]. Fetuin-A participated in the development of diabetes and kidney disease [90]. 

Fetuin-A is able to bind to the extracellular portion of the transmembrane β-subunit of the insulin receptor (InsR). Fetuin-A, through its interaction with insulin, has a significant effect on glucose homeostasis. Only two proteins can interact with the extracellular part of InsR: insulin and Fetuin-A. Insulin induces the receptor’s intrinsic tyrosine kinase activity, which is responsible for glucose transport. In contrast, Fetuin-A has the opposite effect and deactivates tyrosine kinase. In that case, insulin signaling is impaired, which is an element of insulin resistance development [91,92]. Fetuin-A also participates in modulating FFA-mediated pancreatic β-cell inflammation, which also increases insulin resistance [93].

Studies have revealed that higher levels of circulating Fetuin-A are positively correlated with the incidence of T2DM and were more prominent in women [94,95]. A diet abundant in fat enhances Fetuin-A expression [96]. Furthermore, a strong correlation exists between Fetuin-A and obesity-related complications, and certain factors, such as weight loss and taking pioglitazone or metformin, can contribute to lower Fetuin-A levels [94,97]. Novel antidiabetic drugs are presented in Table 1.

## 5. The Role of Immunomodulation through Nutrition as a Key Therapeutic Strategy in Patients with Diabetes

**Figure 2:**
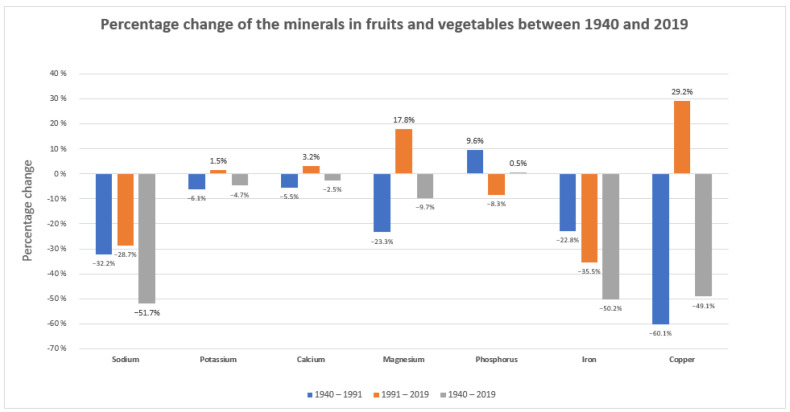
Percentage change in the minerals in fruits and vegetables between 1940 and 2019.

The proper function of the immune system depends not only on genetic factors and concomitant diseases but also whether the building material for the synthesis of its components is provided. For example, metals, which play a key role in virtually all basic biological processes, are crucial components of almost half of all enzymes [9]. Microelements, macroelements, and vitamins are necessary for the proper functioning of the entire immune system. Their deficiency disrupts the functioning of physical barriers and impairs the innate immune response (both cellular and biochemical), inflammatory response, and adaptive response (antigen presentation, humoral, and cell-mediated immunity) [98]. A proper diet should provide all the necessary nutrients and non-nutritive bioactive compounds. Unfortunately, changes introduced in the food industry have resulted in a significant loss of ingredients necessary for the proper performance of the immune system. This is due to, among others, the use of artificial fertilizers containing much less valuable ingredients than natural fertilizers. A decline in essential minerals in fruit and vegetables has been reported in the UK and other countries. A new analysis of long-term trends in mineral content in fruit and vegetables from three editions of the British Food Composition Tables (1940, 1991, and 2019) was carried out. Concentrations of all elements except phosphorus decreased between 1940 and 2019, and the largest overall reductions in this 80-year period concerned Na (52%), Fe (50%), Cu (49%), and Mg (10%) [6]. The analysis is presented in Figure 2. Research conducted by Geigy Pharmaceutical Company comparing the concentration of vitamins and minerals in selected plant-based foods in the years 1985–2002 showed a significant decrease in the concentration of calcium, magnesium, folic acid, vitamin B6, and vitamin C. The greatest reduction occurred in bananas and concerned vitamin B6 (95% loss) [99]. The loss of selected components results from the reduced concentration of specific elements in the soil. This loss is intensified by food storage and processing. For example, thiamine occurs mainly in the bran and germ of wheat grains, and up to 50% of it is lost during milling [100,101]. Fresh leafy vegetables stored at room temperature lose up to 70% of folic acid within 3 days. Maharaj P.P. et al. conducted research examining the effects of cooking and frying on folic acid retention in commonly consumed Fijian dishes vegetables (drumstick leaves, taro leaves, bale leaves, amaranth leaves, fern, okra, and green beans). Folic acid loss varied among vegetables from 10 to 64% when cooking and from 1 to 36% when frying. A greater loss of folic acid was observed during cooking. The content of folic acid in water obtained after cooking various vegetables ranged from approx. 11.9 ± 0.5 to 61.6 ± 2.5 µg/100 mL. Boiling is a better choice for cooking vegetables if folic acid intake is taken into account, provided that the cooking water is drunk along with the vegetables [102]. Human levels of vitamins and trace minerals are no longer adequately supported by low-micronutrient-cultured meats and plant-based products produced within existing agricultural food systems [103]. Unfortunately, the need for additional supplementation is underestimated by clinicians who are not aware of the changes that have occurred in food over the past decades. On the part of patients, supplementation, if it is carried out, is often performed in an uncontrolled manner on its own.

### 5.1. Macroelements

In the case of humans, macroelements are elements whose dietary requirement exceeds 100 mg per day. They constitute not less than 0.01% of the dry weight of each organism. They are necessary for the proper functioning of the human body, and their deficiencies are often associated with unpleasant health consequences.

#### 5.1.1. Magnesium 

The second most common intracellular cation and the fourth most abundant mineral is magnesium (Mg^2+^). This element comes in bonded form and serves a myriad of functions in eminent physiological processes. This element in the human body is found in intracellular space, mostly in bones but also in muscle cells, soft tissues, and organs. Less than 1–2% of Mg^2+^ is also found in blood, and it is present in a three times greater concentration in erythrocytes than in plasma [104]. This mineral, which can be consumed together with fruits, vegetables, seeds, grain, cereals, meat and fish, and berries, is an important cofactor in over 600 activities [105]. The daily allowance of magnesium for females is 320 mg and 420 mg for males [106]. Mg^2+^ is a part of numerous organic substances, like proteins, nucleic acids, and nucleotides. Magnesium management is regulated by intestinal absorption, renal reabsorption/excretion by hormonal control, and the source of magnesium in the intracellular space (bones, etc.) [105]. Increased magnesium excretion takes place in unregulated diabetes and metabolic acidosis. This nutrient’s role is to regulate cell cycle progression, stabilize membrane structure and its potential, participate in DNA and RNA synthesis, and aid nervous system functioning or the secretion of enzymes and hormones [107,108]. Its versatile role also includes oxidative phosphorylation and muscle contraction and glucose, protein, and lipid metabolism. On top of that, Mg^2+^ is essential for ATP production in mitochondria [104,108]. 

Magnesium has a major impact on the regulation of a wide range of immunological processes. It affects the acute phase response and macrophage function. Mg influences the development and proliferation of lymphocytes. It has been found that an adequate amount of magnesium is required for the immune T cells so that they can fulfill their function in combating pathogens properly. Mg^2+^ insufficiency makes the cell more sensitive to oxidative stress in diabetes, which accelerates the development of diabetes-related complications [105]. 

This cation also controls blood glucose and blood pressure. Studies on animals have been carried out and found that dietary Mg consumption (50 mg/mL in drinking water) for 6 weeks lowered glucose levels, ameliorated mitochondrial function, and reduced oxidative stress and declined oxidative stress, as both are two main factors of insulin resistance [109,110].

Some studies show the connection between chronic magnesium deficiency and the occurrence of symptoms, such as overweight and obesity, insulin resistance (IR), and T2DM. In that case, the protective role of Mg^2+^ is to attenuate inflammatory processes, improve glucose and insulin metabolism, and normalize the lipid profile [107]. 

There is a strong cooperation between magnesium and insulin signaling pathway activation. The cation affects the tyrosine kinase (TK) of insulin receptor activity and regulates peripheral insulin sensitivity, while at the same time, insulin regulates magnesium homeostasis. When the concentration of magnesium is decreased, TK activity is impaired, the insulin activity in the cell is blocked, and insulin tolerance rises. The insulin signaling pathway allows for the regulation of glucose transport or glycogen synthesis, and mistakes in this way can lead to decreased insulin transport and glucose uptake, which may cause hyperglycemia. Certain studies demonstrated that a deficiency of Mg^2+^ can cause the development and progression of diabetes. It is said that magnesium deficiency can predict faster deterioration of kidney function, promote atherosclerosis, and increase the changes for diabetic microvascular complications [111]. 

Several studies indicate that dietary Mg intake and a higher risk of T2DM are interconnected [112]. Some of them show that magnesium supplementation improves insulin sensitivity markers and that it has a favorable impact on glucose concentration in diabetics [107].

#### 5.1.2. Calcium

Calcium (Ca^2+^) is the most abundant mineral in the human body. It can be absorbed mainly from the diet with dairy products, dark green leafy vegetables, and calcium-fortified food. The main storage of calcium, which reaches up to 99% in the body, is bones, which that have a structural function, and a residual 1% is in the intracellular and extracellular fluid. Calcium metabolism regulation is based on adequate intestinal calcium absorption, proper storage of calcium in bones, and the excretion of excess calcium by the kidneys, and it is all under the control of the 1,25-dihydroxyvitamin D, parathyroid hormone, and ionized calcium [113]. 

This mineral is versatile since it serves an important function in a wide range of biological processes, such as muscle contraction, blood coagulation, hormone secretion, and neurotransmission [114,115]. Adequate Ca^2+^ consumption may have an impact on the release of harmful substances that may enhance the risk of diabetes [116]. Immune system cellular processes, such as proliferation, division, activation, and gene transcription, may occur because of the calcium signals. An immune response decreases intracellular Ca^2+^ and then activates its flow to increase the intracellular Ca^2+^ concentration, and it occurs due to CRAC channel activation [117]. Diabetes mellitus is a disease that disrupts the normal functioning of organs in the human body, including calcium metabolism, making it difficult for the organs that regulate calcium to function properly. Incorrigible calcium management may disturb regulation processes in blood cells or cardiac and skeletal muscles. Calcium signaling is indispensable for the work of pancreatic β-cells, which secrete insulin in response to increased glucose levels through calcium channels [118]. 

A study involving a 10-year follow-up found that higher dietary calcium consumption was related to reduced risk of diabetes. Another study in 2011 showed that yogurt consumption with high Ca^2+^ content improved fasting glucose and fasting insulin [119]. There are also studies indicating that patients with uncontrolled hyperglycemia have a higher risk of hypocalcemia [109].

#### 5.1.3. Potassium

The influence on insulin secretion is also observed in terms of potassium (K^+^), which is the most abundant cation that is located in 98% of intracellular fluid in mainly muscle cells, and the remaining 2% is in extracellular fluid. K^+^ is mostly found in higher concentrations in fruits, vegetables, and milk. It is recommended to consume more than 3.5 g per day [120]. Potassium participates in maintaining cell function, especially in muscle and nerve cells. Adding salt to food reduces potassium and increases the sodium content. The Food and Nutrition Board of the Institute of Medicine has established the recommended daily intake of K^+^ to be 4700 mg, and for the WHO it is 3150 mg [121]. Normal serum potassium levels are between 3.6 mmol/L and 5.0 mmol/L, and values below are called hypokalemia; it is said that patients with comorbidities, especially diabetics, are exposed to an unfavorable course of treatment [122]. K^+^ concentration in a cell depends on the transport of potassium ions through the Na-K APTase pump and its leakage through the K^+^ channels [123]. 

Potassium plays an important role in immune cells in the human body. K^+^ gradient is required for the cell to maintain a membrane potential for Ca^2+^ gradient. It is needed for immune cell activation, as it happens due to a Ca^2+^ influx in inflammatory gene transcription. The potassium channel leads to the activation of mononuclear cells and nitric oxide production in macrophages [124].

Under physiological circumstances, insulin is released when the concentration of extracellular K^+^ is high. This process occurs through inhibiting the ATP-sensitive potassium channels of pancreatic B cells. In uncontrolled diabetics, changes in potassium concentrations outside the normal limits affect the regulation of glucose renal excretion. Low insulin concentration increases renal elimination of glucose, which increases sodium influx, and this increases K^+^ elimination. Low serum potassium may be a disturbing factor for insulin secretion and cause glucose intolerance or diabetes [122].

Research by Chatterjee R. et al. demonstrated the effects of low potassium intake and low serum potassium levels on decreased insulin sensitivity and increased insulin secretion [125]. In the case of the K^+^ and Ca^2+^ action for the insulin secretion process, its decreased intake has an impact on reduced risk of diabetics. Increased potassium intake may bring good health benefits, like improved glucose control, glucose intolerance, insulin resistance, elevated blood pressure, and hypertension [126]. 

#### 5.1.4. Sodium

The main cation of extracellular fluid that is essential for adequate cell functioning is sodium (Na^+^). To maintain fluid balance and mineral metabolism at the appropriate level, sodium consumption at the appropriate level is necessary [127]. This element takes part in regulating nerve and muscle function. Along with the increased consumption of processed foods, salt intake is increased as well. Despite the fact that there are many negative effects of increased salt consumption, it may protect against dehydration. The WHO recommends a daily sodium intake of 2000 mg per day [128]. 

Sodium has an immunostimulatory role in the immune system. Na^+^ can stimulate immune cells to trigger a stronger immune response [129].

Research by Ming L. et al. shows that higher daily sodium consumption is connected with an increased risk of diabetes, and the risk gets higher by 1.20 times for every 1 g Na^+^ consumed [128]. 

Research conducted by Suckling RJ et al. showed that reduced sodium intake did not affect insulin sensitivity, fasting glucose, or insulin concentration [130]. Another study showed that limiting sodium intake improves insulin resistance because of the increased secretion of adiponectin and diminution of pro-inflammatory processes. A high concentration of sodium in the diet increases cortisol secretion and insulin resistance, and despite this Na^+^ still can improve the nervous system in diabetics [110]. 

#### 5.1.5. Phosphorus

Phosphorus (P) is another essential macronutrient that consists of up to 1% of the total human body [131]. The recommended daily intake of phosphorus ranges from 550 to 700 mg [132]. The Institute of Medicine set the maximum recommended phosphorus daily diet intake to 4000 mg. It is the element that humans consume by eating fast food and restaurant meals rich in phosphate but also mild and dairy products, fish and meat, and grain products. Phosphorus management in the human body is hormonally regulated by fibroblast growth factor 23 (FGF-23) and parathyroid hormone (PTH) by their effects on the production and concentration of vitamin D, which regulate bone metabolism and the intestinal absorption of calcium and phosphorus, which is absorbed in 55–80% of cases [131]. Phosphorus is one of the main building components of cell membranes and nucleic acids. Its role is mostly confined to bone mineralization, energy generation, and regulating acid–base homeostasis in order to maintain normal pH. A high concentration of FGF-23, the hormone that regulates the phosphorus economy, is connected with insulin resistance. High daily phosphorus intake may be a factor in secondary hyperparathyroidism and bone loss and can have a destructive influence on the cardiovascular system. Recently, the consumption of processed foods has increased, as has the incidence of diabetics, and there is research that shows the connection between the higher phosphorus consumption and the higher risk of diabetics [132].

#### 5.1.6. Sulfur

The essential element sulfur is absorbed from the digestive tract in the form of amino acids. Two main amino acids (SAAs) are methionine and cysteine, and it is essential to the synthesis of compounds involved in the metabolic process and also a component of antioxidants involved in the body’s defense mechanisms [133]. The sources of sulfur are mostly vegetables, such as broccoli, cauliflower, garlic, and onion. The protective effect of sulfur is based on its influence on the cardiovascular system, glucose, and anti-inflammatory effect. Protein intake and diabetes depend on its type and quantity [134]. One of the protein sources is animals, such as red meat or processed meat, and the other from plants, such as nuts and soy. One of the studies shows the important correlation between consuming animal proteins and the higher risk of diabetics and demonstrates that people who had more plant protein in their diet and reduced their red meat consumption to 30% had a lower risk of getting diabetes [135]. Decreased consumption of SAAs may improve the insulin signaling pathway, which may have a beneficial effect on insulin sensitivity and glucose metabolism [134]. An SAA through its participation in the synthesis of antioxidants, especially glutathione, has an oxidative role. There is a study that shows that the consumption of cysteine increases the glutathione level, and it may have a good influence on glucose levels in the blood [136]. Limiting SAA intake may have a beneficial effect on preventing insulin resistance and might lower the glucose and glycated hemoglobin levels [137].

### 5.2. Microelements

Microelements are trace elements necessary to maintain the appropriate function of the organism. Although the demand for them is small (compared to the demand for macroelements), they are requisite for the proper development and maintenance of life functions.

#### 5.2.1. Zinc

Zinc is a crucial microelement in metabolism. It is considered to control over 100 enzymes that are responsible for substantial processes, such as protein folding, gene expression, cell signaling, and cellular processes, including cell division and apoptosis. In terms of the immune system, zinc is known to strongly affect factors of an immunological response, such as chemokines, pro-inflammatory cytokines, complement factors, and bacterial wall compounds. This leads to the early activation of polymorphonuclear leukocytes, which together with macrophages, are listed among first responders when infection occurs. Both zinc deficiency and zinc excess are undesirable because they are proven to inhibit nicotinamide adenine dinucleotide phosphate oxidases, whose activity is responsible for killing pathogens. This process takes place after phagocytosis, which is also influenced by the accessibility of zinc. This microelement can positively influence the amount of cytokines and act as an antioxidant [109,138].

Due to the various ways in which zinc affects many signal pathways, it is not fully understood how exactly the lack of zinc homeostasis contributes to the development of T2DM. It has been proven that zinc transporter 8 (ZnT8) plays an important part in zinc uptake by insulin secretory granules in beta cells [139,140]. In a study conducted in vivo on mice, it was demonstrated that ZnT8 is essential for beta-cell zinc influx, glucose-stimulated insulin secretion, and insulin processing, as well as the formation of insulin granules [140,141]. Another transporter that is considered important in the pathomechanism of the disease is ZIP7. It is a transporter that is involved in the process of endoplasmic reticulum (ER) stress. ER is critical for the correct processing and folding of proteins, and ER stress arises when the folding capacity of the ER is outpaced by the influx of nascent, unfolded polypeptide chains. Cells undergoing this type of stress will activate a UPR (unfolded protein response) pathway, which will decrease the transcription of genes that encode secretory proteins, causing decreasing folding demand on the ER. ZIP7 is a transporter located in an early secretory pathway, including ER controlling the movement of zinc from this subcellular organelle into cytosol. The importance of ZIP7 is emerging as a key transporter implicated in maintaining ER homeostasis. In that way, zinc protects the ER from imbalance and, therefore, it helps avoid the reduced transcription of genes that are responsible for secretory proteins [142]. 

#### 5.2.2. Selenium

Selenium is a microelement and plays a distinctive role in our immune system. It is indispensable for the function of selenoproteins, which act as redox regulators of several key enzymes, transcription factors, and receptors, and, therefore, they affect the functioning of leukocytes and NK cells. Selenoproteins function as well as cellular antioxidants [10]. Selenium influences innate immunity as well as adaptive immunity. Innate immunity regulates the viability of NK cells, macrophages, DCs, granulocytes, mast cells, and microglia. Selenium is also proven to promote the proliferation of T cells, promote CD4+ T cell differentiation into Th1 cells, and suppress the activity of cellular 5-lipoxygenase, which all are mechanisms enhancing adaptive immunity [143].

Many factors are said to influence the function of pancreatic islets and contribute to their metabolic dysfunction. Some of these factors are chronic low-grade inflammation, redox imbalance, mitochondrial dysfunction, and endoplasmic reticulum stress. During the development of T2DM, M1-like macrophages become the most abundant immune cells in crucial tissues, such as visceral white adipose tissue, liver tissue, and pancreatic islets. The inflammation of these tissues is a key component of developing beta cell dysfunction, and M1-like macrophages happen to be a primary source of pro-inflammatory cytokines. This process leads to the accumulation of ectopic lipids in beta cells. Moreover, H_2_O_2_ (ROS), which is required for proper insulin biosynthesis, secretion, and signaling, when imbalanced, can lead to oxidative stress, resulting in disruption in signaling pathways and even death of the cell. Taking into consideration that selenoproteins, such as glutathione peroxidases, are responsible for removing H_2_O_2_, selenoproteins S are involved in ER function, and a group of selenoproteins called thioredoxin reductases control redox function. It is safe to say that the imbalance of selenium may contribute to developing T2DM, but the exact mechanism remains unclear [144]. More studies have shown a positive correlation between high levels of Se and the presence of T2DM [145]. This correlation can be caused by the fact that high Se exposure might affect the expression of key regulators of glycolysis and gluconeogenesis. This action is potentially mediated by selenoprotein GPx-1, and it was demonstrated that the overexpression of this selenoprotein causes insulin resistance [146,147]. 

#### 5.2.3. Iron

Iron is an essential dietary mineral used to support vital human functions, such as erythropoiesis and cellular energy metabolism [108]. It also has a significant influence on our immune system. Iron affects both the innate and adaptive immune system. Its role is to regulate macrophage polarization, but it also partakes in the functioning of neutrophils. Iron is involved in the formation of neutrophil extracellular traps. It is also important in the development, proliferation, activation, and function of NK cells in viral infection [148]. Recent studies proved that adaptive T cell immunity requires serum iron by TfR1 (CD71). In terms of B cell function, it has been proven that the dysfunction of these cells can happen due to the mutation of transferrin receptor 1 (TfR1) encoded by TFRC and can lead to immunodeficiency [149,150]. 

High iron is a risk factor for T2DM and affects most of its cardinal features, such as decreased insulin secretion, insulin resistance, and increased hepatic gluconeogenesis [151]. One possible mechanism underlying this relationship might be that elevated serum ferritin can interact with other kinds of pathogenic factors, impair the function of islet beta cells, affect the secretion of insulin, and increase the risks of T2DM [152]. Another explanation might be that because insulin is involved in regulating the transcription of serum ferritin and increasing the use of iron in peripheral tissues, when there is too much iron, insulin secretion is affected and the overload of iron causes oxidative stress [153]. This phenomenon is caused by an increase in iron-catalyzed hydroxyl radicals, which leads to systemic insulin resistance and hyperglycemia [154]. Iron is a powerful pro-oxidant and may cause cellular damage in the mechanism of producing reactive oxygen species, and taking into consideration that beta cells in the pancreas are particularly susceptible to oxidative injury, it can be another way in which excess iron leads to T2DM [155]. Serum ferritin is also considered as an acute-phase marker; therefore, every inflammation causes an imbalance in insulin secretion [153].

Although the exact molecular mechanism of iron-regulated pathology in diabetes remains vague, many studies over the years have proven that there is definitely a dependence between excess body iron and the presence of T2DM [155].

#### 5.2.4. Iodine 

Iodine in humans is primarily scrutinized for its effects on the thyroid gland. There is some evidence indicating that iodine could also influence the function of other organs. Iodine is suggested to work for the benefit of the immune system by neutralizing ROS and making cell membranes less reactive to free radicals. I2 acts as a scavenger of reactive oxygen species, like hydroxyl radicals or superoxide anions, generating neutral components of hypoiodous acid or hydroiodic acid. Iodine, in combination with arachidonic acid, forms iodolipid 6-iodolactione (6-IL), which suppresses the activity of pro-inflammatory enzymes, like nitric oxide synthase and cyclooxygenase type 2 [156].

Iodine in excessive amounts diminishes cell viability and compromises the function of insulin secretion in islet beta cells [157]. Thyroid function is important for regulating metabolism, and abnormal thyroid function can have substantial effects on blood glucose control in diabetes [109]. A study conducted by O S Al-Attas et al. showed that the concentration of urine iodine was significantly lower in T2DM than in healthy control subjects. The decreased levels of iodine concentration in T2DM patients may have deleterious effects on metabolic functions [158]. An alternative study that showed a correlation between levels of iodine and T2DM was the study conducted by Cuneyd Anil et al., which resulted in the conclusion that the TSH level of the diabetic patients was higher than in the control group and so was the mean thyroid volume [159].

#### 5.2.5. Copper

Copper is a trace element found mainly in the liver, bones, and muscles. It carries particular importance in the functioning of the immune system. As a redox active metal, copper is the ideal cofactor for enzymes involved in electron transfer and oxygen chemistry [160]. Roughly 30 metalloproteins are dependent on the accessibility of copper, and their tasks range from respiration (cytochrome c oxidase; COX) to free radical detoxification (superoxide dismutase; SOD) [160,161]. There is also ceruloplasmin, which is a multicopper oxidase. Its main role is to oxidize Fe^2+^ to Fe^3+^, yet it is additionally an acute phase protein induced in response to inflammation, trauma, or infection [160,162]. A possible explanation as to why the amount of multicopper oxidase increases during infection might be that ceruloplasmin helps deliver copper to sites of infection to combat pathogens with copper toxicity. Moreover, it has been shown that the enrichment of plasma Cu levels could enhance both innate and adaptive immunity in humans in the context of its antiviral activity, which can serve preventively and therapeutically against COVID-19 [149]. Cu is involved in the function of T-helper cells, B cells, neutrophils, NK cells, and macrophages [163].

The results of different studies are inconsistent concerning the association between copper consumption and the likelihood of developing diabetes. The result of a cohort study performed by Eshak et al. on 16,160 Japanese patients showed that those who had a higher intake of copper and iron had a greater risk of developing T2DM [164].

Different cohort studies conducted by Cui et al. evaluated the diet of 14,711 adults and managed to associate dietary copper with a higher risk of developing T2DM. The study was based on people who had neither diabetes, hypertension, nor any cardiovascular diseases and took part in the China Health and Nutrition Survey [165]. One possible way of explaining the way copper influences the presence of T2DM is that copper is an essential component of the enzyme copper/zinc superoxide dismutase (Cu/Zn SOD), which helps in the clearance of free radicals that accumulate in cells as a result of metabolic stress. Another mechanism might be that copper is involved in the reactions of glutamic acid decarboxylase (GAD), which is a major beta-cell antioxidant that is altered by reactive oxygen species (ROS). Anti-GAD antibodies contribute to the pathogenesis of T2DM [166,167]. Copper in high doses is suggested to have a pro-oxidant activity, so it is also possible for copper to cause the production of ROS through the Fenton reaction, which can hinder many processes, including ones associated with insulin resistance development and impaired glucose metabolism [168].

#### 5.2.6. Cobalt

Cobalt is mainly known for its role as a metal constituent of vitamin B12 [169]. Subsequent sections of this manuscript will elucidate and expound on the topic of vitamin B12′s role in the immune system. Agata Szade et al. showed that cobalt protoporphyrin IX (CoPP) increases plasma concentrations of granulocyte colony-stimulating factor (G-CSF) IL-6 and MCP-1 in mice, triggering the mobilization of granulocytes and hematopoietic stem and progenitor cells (HSPCs). Compared with recombinant G-CSF, CoPP does not increase the number of circulating T cells [170].

Research on the role of cobalt in the human immune system is currently limited. Studies mostly concentrate on the toxicity of inorganic cobalt and how it evokes a chain of changes in cells. In some cases, it can lead to an immunological response known as a type IV hypersensitivity reaction [171].

The studies performed on human subjects in order to assess the levels of cobalt in diabetic patients are inadequate. Saker et al. proved that cobalt chloride decreased gluconeogenesis in diabetic rats through its glucose-lowering effect [109,172]. Cobalt treatment showed amelioration in nephropathy and heart function in a rat model of T2DM by alleviating oxidative stress [109,173].

#### 5.2.7. Chrome

Chrome is an essential micronutrient for humans. The reduction in Cr(VI) to Cr(III) leads to the generation of reactive intermediates, which, in conjunction with oxidative stress, induces tissue damage. This initiates a cascade of cellular events, including the modulation of the apoptosis regulatory gene p53, contributing to the cytotoxic, genotoxic, and carcinogenic effects associated with Cr(VI)-containing compounds. Conversely, chromium serves as an essential nutrient crucial for insulin action in body tissues, facilitating the utilization of sugars, proteins, and fats. Chromium plays a pivotal role in modulating the immune response through either immunostimulatory or immunosuppressive processes, as evidenced by its effects on T and B lymphocytes, macrophages, cytokine production, and immune responses, which may elicit hypersensitivity reactions [174]. 

Chrome has been found to electively improve glucose tolerance by reducing insulin resistance [109]. A study based in China showed that supplemented Cr improved the blood glucose, insulin, cholesterol, and HbA1C levels of patients with T2DM in a dose-dependent manner [109,175]. Chromium improves the glucose/insulin levels in subjects with hypoglycemia, hyperglycemia, and diabetes with no detectable effects on the control group. Cr also improves insulin binding, receptor number, and the functioning of beta cells [109,176]. 

Rajendran et al. concluded the relationship between serum Cr levels and T2DM. A decrease in Cr levels occurred in response to oxidative stress in T2DM patients. In their study, patients with uncontrolled glucose levels with T2DM demonstrated lower levels of serum Cr levels compared to the control group. The HbA1c and serum Cr levels were inversely correlated in a statistically significant way [177]. The exact mechanism has not been thoroughly studied. Further research into this relationship could definitely be beneficial in the future. 

#### 5.2.8. Manganese

Manganese is a trace element present in a variety of physiological processes, such as antioxidant defenses, reproduction, and neuronal function [178]. Manganese is incorporated into a number of metalloenzymes, like Mn superoxide dismutase (SOD Mn^2+^), glutamine synthetase (GS), pyruvate carboxylase, and arginase [179]. Mn^2+^ is required for the host defense against DNA viruses by increasing the sensitivity of the DNA sensor cGAS and its downstream adaptor protein STING. Mn^2+^ is released from mitochondria and the Golgi apparatus upon virus infection and accumulated in the cytosol where it is bound to cGAS, enhancing the sensitivity of cGAS to dsDNA and its enzymatic activity. On this pathway, manganese supports antitumor immunity. Mn^2+^ is also engaged in the innate immune detection of tumors, as Mn-insufficient mice had significantly enhanced tumor growth and metastasis and greatly reduced tumor-infiltrating CD8+ T cells in a cGAS-STING-dependent manner [180].

Some studies concerning manganese and its influence on diabetes may be found; however, they are ambiguous. Adewuni et al. showed that there is a significantly lower serum concentration of Mn in diabetic patients compared to the control group [181]. On the contrary, Anetor et al. demonstrated that mean serum Mn concentration in patients with T2DM was higher than in the control group [182]. The mechanism by which manganese influences the development of T2DM is not known. One possible explanation might be that intracellular Mn^2+^ accumulates predominantly in mitochondria, where it may interfere with an electron transport chain and the oxidative phosphorylation process, causing a significant formation of ROS. The direct involvement of manganese in the electron transport chain may lead to suppressed ATP production, increased leaked electron flux, and the formation of highly reactive and detrimental hydroxyl radicals [183]. While the initial exploration of the topic is insightful, further research would enhance its usability and provide a more comprehensive insight. 

#### 5.2.9. Molybdenum 

Molybdenum is a trace element that is important for various enzymatic processes. It needs to be complexed by a special cofactor to gain catalytic activity [184]. There are four molybdenum enzymes known in humans, each catalyzing either catabolic or detoxifying reactions [185]. All of these enzymes share the same molybdenum cofactor (Moco). For example, sulfite oxidase (SOX) catalyzes the terminal step in oxidative cysteine catabolism, the oxidation of sulfite to sulfate. SOX links sulfite oxidation to the reduction in cytochrome c [186]. The existing literature does not extensively cover the topic of molybdenum’s role in the immune system. A study on calves was conducted, with the objective to determine the effects of dietary Cu and Mo on immune function and other features. It was concluded that claves that were fed with the supplemental Mo had lower antibody production than the calves supplemented with Cu, which suggests that Mo supplementation without the supplemental Cu induces a more severe functional Cu deficiency [187].

#### 5.2.10. Silicon

The available scientific literature on the influence of silicon on the immune system is notably scarce, with a paucity of comprehensive data addressing this specific relationship. The study by Liangjiao et al. showed that silica nanoparticles (SiNPs) can potentially have a toxic influence on the immune system. The main mechanisms were pro-inflammatory responses, oxidative stress, and autophagy. SiNPs cause oxidative stress by increasing membrane lipid peroxidation and the levels of ROS and by decreasing intracellular glutathione levels (GSH) [188].

### 5.3. Vitamins

Vitamins are low-molecular-weight organic compounds whose presence in the body in small amounts is necessary for the proper management of sundry metabolic processes. For many organisms, including animals and humans, these compounds are exogenous and must be supplied with comestibles. Vitamins do not have nutritional or energy functions, yet they serve a regulatory function, acting as biocatalysts of metabolic processes in cells. However, without these substances, it would be impossible for the body to function properly. A great deal of studies have proven the role of vitamins in modulating the inflammatory response and behavior balance between immune cells or inhibiting the production of cytokines [189]. Due to the immunomodulatory role of vitamins, numerous studies are conducted to investigate their impact on supplementation for diabetes by monitoring glucose levels, pancreatic β-cell function, and glycated hemoglobin level HbA1c. Many studies have found vitamin deficiencies in the course of diabetes, including vitamin A, vitamin D, or vitamin E [190,191,192].

#### 5.3.1. Vitamin D

Vitamin D is a steroid hormone derived from cholesterol. The largest source of vitamin D in our body comes from its synthesis in the skin under the influence of ultraviolet light; for this reason, a multitude of factors influence the synthesis of vitamin D, e.g., skin pigmentation, latitude, lifestyle, and season. There are several forms of vitamin D-D2 (ergocalciferol) and D3 (cholecalciferol). Vitamin D is transported in its inactive form through the DBP-binding protein to the liver, where it is converted into 25-hydroxyvitamin D (25(OH)D). The active form of vitamin D–1,25-dihydroxyvitamin D–1,25(OH)2D (calcitriol)—penetrates target cells and binds to the nuclear receptor vitamin D (VDR). Vitamin D bound to nuclear receptors acts directly on the DNA of cells, causing the production of special proteins. One of the pivotal functions of vitamin D is its participation in the metabolism of calcium and phosphate, as well as bone homeostasis. Additionally, vitamin D affects many other physiological processes in the body through VDR receptors. One of them is the effect of vitamin D on cells of the immune system, where the presence of the VDR receptor was proven in almost all cells of the aforementioned system, and VDR receptor polymorphism has been associated with an increased frequency of autoimmune diseases [193].

Vitamin D exerts its effects on both the innate and adaptive immune systems through the VDR. One of the types of cells that contain the VDR receptor are antigen-presenting cells (APCs), T lymphocytes, and macrophages [194,195]. Calcitriol stimulates the differentiation and activation of macrophages. It promotes their antimicrobial activity by increasing chemotaxis and phagocytosis by stimulating the local production of defensins (e.g., cathelicidin and β2-defensin). Calcitriol reduces the ability of macrophages to present antigens and stimulates T cells by reducing the surface expression of MHC class II molecules [193]. Additionally, calcitriol promotes the induction of immune tolerance through its anti-inflammatory effects. It inhibits the differentiation, maturation, and function of dendritic cells (DCs), making them incapable of acting as mature APCs, which allows the development of immunological tolerance [196,197].

The effect of vitamin D on the immune system has contributed to research on the impact of vitamin D supplementation on the course of diseases related to the immune system, for example, type 1 diabetes. It has been studied that it exerts an anti-inflammatory effect directly by affecting the cells of the immune system, thereby weakening the destruction of pancreatic β-cells, which produce insulin. The destruction of pancreatic β-cells by autoantibodies underlies the pathophysiology of type 1 diabetes. Vitamin D has the potential to restore immune tolerance, which counteracts the autoimmune response and slows or stops the progression of the disease by preserving residual β-cell mass and function and improving glycemic control [193,198,199]. Vitamin D acts through the VDR receptor in the pancreas and regulates insulin secretion in the pancreatic islets. It also affects insulin sensitivity in many peripheral metabolic organs. In type 1 diabetes, pancreatic β-cells are destroyed by autoantibodies. β-cell-specific autoantigens (such as insulin, pro-insulin, and IGRP) are presented by antigen-presenting cells (APCs), triggering cytotoxic T cell responses that cause β-cell damage. It has also been proven that autoantigens are presented to APCs by macrophages [200]. For this very reason, the effect of vitamin D based on reducing the presentation of antigens to APCs by macrophages and inhibiting the differentiation of dendritic cells into APCs could reduce the inflammation occurring in type 1 diabetes. Additionally, patients with type 1 diabetes have lower levels of vitamin D. Studies have shown that a larger number of diabetic patients have autoantibodies against DBP, which could explain the reduced vitamin D concentration in people with diabetes.

In the case of vitamin D supplementation, the need for insulin was reduced and so was the risk of diabetes. After starting supplementation, there was an initial increase in C-peptide concentration. The level, however, did not persist and returned to the level of the control group that did not consume supplementation. Some studies also found lower HbA1c levels after starting vitamin D supplementation, which could correlate with better baseline β-cell function because the HbA1c lowering effect was lost over time. For this reason, it was not possible to determine whether the improvement in test results was due to vitamin D supplementation itself or to individual differences in the course of the disease [193,201]. The conducted research leads to the conclusion that supplementation is beneficial mainly at the early stage of diagnosis, as it reduces the need for insulin and the risk of complications related to the disease [192,198,201]. Studies have also been conducted that clearly demonstrate the benefits of vitamin D supplementation in people with type 1 diabetes. Patients have lowered serum glucose and HbA1c levels [202].

The impact of vitamin D supplementation in patients with type 2 diabetes remains invaluable. Supplementation of this vitamin significantly improves the concentration of serum triglycerides, LDL cholesterol and total cholesterol, insulin, HbA1C, and HOMA-IR. It also significantly reduces the concentration of highly sensitive C-reactive protein (hs-CRP). Low vitamin D levels are strongly associated with insulin resistance, impaired insulin secretion, and an increased risk of T2DM cases, especially in people at high risk of T2DM. Vitamin D deficiency is closely associated with various micro- and macrovascular complications of T2DM, including peripheral neuropathy, erectile dysfunction, retinopathy, diabetic kidney disease, and overall mortality. Interventional studies in people with T2DM and CKD have shown significant improvements in kidney function, especially when vitamin D analogs were combined with RAAS inhibitors [203].

#### 5.3.2. Vitamin E

Vitamin E is the term for a group of tocopherols and tocotrienols, of which alpha-tocopherol has the highest biological activity. It cannot be synthesized in the human body, so it can only be obtained from dietary nutrients, such as olive oil, almonds, sunflower oil, hazelnuts, sprouts, and grain germs. Vitamin E is an essential nutrient for reproduction [204]. Its functions are primarily based on antioxidant activity, i.e., it has the ability to scavenge free radicals, inhibit lipid peroxidation, and chelate transition metal ions. Recent discoveries have shown that vitamin E has various potentially beneficial effects on human health, such as antiallergic, anti-atherosclerotic, anticancer, antidiabetic, antilipidemic, antihypertensive, and anti-inflammatory; furthermore, it prevents the development of obesity and is neuroprotective [205].

Vitamin E mainly serves as an antioxidant, reducing the accumulation of lipid peroxides and free radicals. Its effect on the immune system is based on the inhibition of the activity of cyclooxygenase 2 COX2. By affecting COX2, it reduces the production of prostaglandin E2 PGE2, which is involved in the body’s inflammatory response. Vitamin E activates T cells and modulates the balance between Th1 and Th2 lymphocytes. Vitamin E reduces the inflammatory response by reducing the production of pro-inflammatory cytokines, such as TNF-α, IL-1, and IL-6, by peripheral blood mononuclear cells [189]. Higher concentrations of the vitamin inhibit the formation of 5-hydroxy-eicosatetraenoic acid 5-HETE and 5-hydroperoxy-eicosatetraenoic acid 5-HPETE in human neutrophils, suggesting an inhibitory effect of vitamin E on 5-LOX 5-lipoxygenase. By blocking 5-LOX, they prevent the transformation of arachidonic acid into leukotrienes, which inhibits the inflammatory response pathway in the body. In addition to the inflammation data, long-chain vitamin E has been shown to have cytotoxic potential in several cancer cell lines. Its pro-apoptotic activity has also been observed in human macrophages [205].

A study by Jin Z et al. showed that vitamin E could effectively reduce urinary microalbumin, urinary albumin excretion rate, and serum nitric oxide levels in patients with type 2 diabetic nephropathy [206]. A study by Mehvari F et al. revealed that vitamin E concentrations were lower in patients with diabetes and coronary artery disease (CAD) compared to diabetic patients without CAD. The results of this study confirm that oxidative stress may be an important factor in increasing the susceptibility of some diabetic patients to CAD. Increased oxidative stress may be a potential therapeutic target for the prevention and treatment of CAD in diabetic patients [207].

#### 5.3.3. Vitamin C

Vitamin C, or ascorbic acid, is a water-soluble vitamin. It is a compound that cannot be synthesized in the body, so the main source of vitamin C for humans is diet. Due to its solubility in water, it can easily lead to hypovitaminosis, so it is mandatory for humans to supplement it systematically with diet. Vitamin C is found in citrus fruits, such as orange, kiwi, lemon, guava, and grapefruit, and vegetables, such as broccoli, cauliflower, Brussels sprouts, and peppers.

The main feature of vitamin C is its ability to donate electrons; it is a strong antioxidant and additionally acts as a cofactor for biosynthetic and gene-regulating enzymes. It also affects the functioning of the immune system by acting on cell functions of both the innate and adaptive immune systems. Vitamin C supports the epithelial barrier function against pathogens. It supports the removal of oxidants through the skin, protecting it from environmental oxidative stress. Vitamin C accumulates in phagocytic cells, such as neutrophils. Within cells, it promotes chemotaxis, phagocytosis, and the production of reactive oxygen species. Moreover, it reduces necrosis and potential tissue damage by promoting apoptosis and the removal of neutrophils by macrophages from infected sites. Additionally, vitamin C has been documented to increase the differentiation and proliferation of B and T cells [208]. Recent in vitro experiments investigated the inhibitory effect of vitamin C on the expression of pro-inflammatory mediators, including IL-6 and TNF-α, in blood cells. Additionally, the antioxidant function influences the innate and adaptive immune response [189].

Vitamin C may reduce insulin resistance and cardiovascular complications in patients with T2DM [209]. It plays an inhibitory role in the production of protein oxidation biomarkers, such as advanced oxidation protein products (AOPPs) and advanced glycation end products (AGEs) [210]. Due to the similar structure of vitamin C to glucose, it can be replaced by glucose in response to chemical reactions and prevent the non-enzymatic glycosylation of proteins [211].

#### 5.3.4. B vitamins

##### Vitamin B1

Thiamine is a water-soluble vitamin, also known as vitamin B1. Vitamin B1 is not synthesized in the human body, so it must be obtained through diet. It is found in the vast majority of foods, including yeast, beef, beans, lentils, and nuts. The most biologically active form of thiamine is thiamine pyrophosphate TPP, which acts as a cofactor for two enzymes in the oxidative pathways after glycolysis of the pyruvate dehydrogenase complex and the α-ketoglutarate dehydrogenase complex. TPP is also involved in regulating metabolism in the brain and in the structure and function of nerves [212]. Vitamin B1 also has an antioxidant function, which protects sulfhydryl groups on the surface of neutrophils against oxidative damage. Therefore, thiamine inhibits the oxidative stress-induced stimulation of the nuclear factor kappa-light-chain-enhancer of activated B cell NF-κB. NF-κB is associated with the development of inflammation, autoimmune diseases viral infections, and even the improper development of the immune system. Vitamin B1 also influences the formation of pro-inflammatory cytokines in macrophages [189].

Decreased levels of vitamin B1 in people with T2DM have been established. A fat-soluble thiamine derivative called benfotiamine is effective in raising the blood levels of thiamine compared to water-soluble thiamine derivatives. Benfotiamine reduces glucose toxicity caused by hyperglycemia in T2DM by activating glucose metabolism and insulin synthesis. It also plays a role in blocking pathways responsible for hyperglycemia-induced damage, such as the hexosamine pathway, the formation of advanced glycation end products, and the activation of protein kinase C. It also acts by activating transketolase (TK), the rate-limiting enzyme of the non-oxidative branch of the pentose phosphate pathway [213].

##### Vitamin B6

Vitamin B6 is a collection of six different forms—pyridoxine (PN), pyridoxal (PL), pyridoxamine (PM), and related 5′-phosphate derivatives. Vitamin B6 is not synthesized in the human body and is obtained through a diet containing potatoes, bananas, nuts, and fish, especially fatty fish, such as salmon and tuna. The biologically active form of vitamin B6 is pyridoxal 5′-phosphate PLP. It prompts cellular metabolism, participating as a coenzyme in up to 150 different enzymatic reactions, such as the synthesis, transformation, and degradation of amines, as well as the biosynthesis and degradation of neurotransmitters. It is not classified as an antioxidant compound and it also affects reactive oxygen species and prevents the formation of advanced glycation end products (AGEs), which are involved in the processes of aging and diabetes [214].

The effect of vitamin B6 on the immune system is based on inhibiting the activation of the NOD inflammasome, LRR, and pyrin domain-containing protein 3 (NLRP3), which results in a reduction in the secretion of IL-1 production. The function of IL-1 is fundamental to innate immunity and the development of acute and chronic inflammation. Inhibiting the production of IL-1 leads to a reduction in the inflammatory response in the organism. Additionally, it has been proven that the administration of PLP significantly reduces the production of mitochondrial reactive oxygen species [189].

Epidemiological and experimental studies have shown an obvious inverse relationship between vitamin B6 levels and diabetes, as well as a clear protective effect of vitamin B6 on diabetes complications [214]. The effect of vitamin B6 on diabetes is related to various processes for which the vitamin is responsible. In vitro experiments on pancreatic perfusion led to the observation that in the case of pyridoxine deficiency, the secretion of insulin and glucagon is impaired [214]. Low vitamin B6 concentration is associated with the occurrence of cardiovascular diseases in people with T2DM, and this relationship may be mediated by plasma fibrinogen and CRP concentrations [215].

Additionally, it has been discovered that the reduction in mitochondrial reactive oxygen species production caused by vitamin B6 supplementation results in a reduction in fasting blood glucose and HbA1c and further improves glycemic control in diabetic patients [214].

##### Vitamin B12

Vitamin B12 is a microelement that humans cannot produce on their own and must be assimilated from animal proteins. It can be found in products, such as kidneys and livers (veal, beef, poultry), fish, mussels, and other seafood, eggs, and dairy products. Vitamin B12 is essential for cell function due to its key role in DNA stability. Vitamin B12 deficiency has been proven to lead to indirect DNA damage, and vitamin B12 supplementation can reverse this effect [216]. It also plays a fundamental role in the functioning of the central nervous system at all ages. With the involvement of folic acid, it participates in the conversion of homocysteine to methionine via methionine synthase, which is necessary for nucleotide synthesis and genomic and non-genomic methylation [217]. Moreover, vitamin B12 has antioxidant properties, which prevent damage caused by reactive oxygen species. DNA protection against reactive oxygen species is based on the removal of free radicals and the reduction in oxidative stress [216]. The impact of vitamin B12 on the immune system involves preserving the equilibrium between CD8+ and CD4+ T cells [189].

Reducing vitamin B12 levels in patients with T2DM is one of the side effects of metformin use [218]. Clinical manifestations of metformin-induced vitamin B12 deficiency include hematological manifestations of megaloblastic anemia and neurological complications, i.e., peripheral neuropathy, spinal cord degeneration, and cognitive impairment [219].

##### Folic Acid

Vitamin B9, renowned as folic acid, is a vitamin obtained mainly from diet. Folate is present in animal tissues, leafy vegetables, legumes, and nuts. Folic acid participates in various remarkable processes in the body, primarily cell replication through enzymatic activity in the synthesis of purine bases for DNA. Noteworthy it is also a transamination cofactor in the conversion of amino acids, especially homocysteine to methionine [220].

In addition to its main functions, folic acid affects the immune system. Several studies have demonstrated its effect on Treg cells [189,221]. Folic acid has a positive effect on the proliferation of T lymphocytes, as it increases phagocytosis and the production of immunoglobulins [189]. Folic acid deficiency also depletes DC cell maturation and impairs CD4+ T cell differentiation. In turn, the administration of high doses of folic acid reduced the inflammatory response by reducing the secretion of pro-inflammatory cytokines IL-4, IL-5, IL-9, IL-13, IL-17, and IL-3. Folic acid also has a large impact on processes related to vitamin B12. Research has proven that maintaining the balance between these vitamins is important in relation to the immune response. It has been shown to have a particular effect on NK cells and cytotoxic CD8+ lymphocytes [221].

Folic acid and vitamin B12 in the course of diabetes mainly affect the level of homocysteine. Its high level in people with type 2 diabetes promotes the development of atherosclerosis. Vitamin supplementation reduces homocysteine levels. Due to the significant role of folic acid in cell replication, it has also been investigated that DNA damage resulting from oxidative stress in diabetes can be reversed by folic acid supplementation. Thanks to this, folic acid reduces the level of endothelial dysfunction in diabetic patients [222,223]. Additionally, folic acid supplementation improved laboratory results in diabetic patients. It improved glycemic control by reducing fasting blood glycosylated hemoglobin, serum insulin, and insulin resistance, as well as homocysteinemia in patients with type 2 diabetes [220,224].

It has been proven that folic acid affects the concentration of CRP, which is one of the inflammatory markers. It is likely that the effect of folic acid on Treg lymphocytes, which are responsible for suppressing excessive immune response, thus contributing to maintaining the homeostasis of the immune system, contributes to the reduction in CRP levels in patients with type 2 diabetes who were supplemented with folic acid [225].

### 5.4. Selected Substances with Immunomodulatory Effects

#### 5.4.1. Coenzyme Q10 (CoQ10)

Persistent hyperglycemia causes oxidative stress due to increased production of ROS, which has been proposed as the root cause underlying the development of insulin resistance, β-cell dysfunction, impaired glucose tolerance, and T2DM [226]. It has also been implicated in the progression of long-term diabetes complications, including microvascular and macrovascular dysfunction. An overabundance of calories through food intake, combined with an inactive way of life in people with T2DM, leads to glucose and fatty acid overload, resulting in the production of ROS. This initiates a chain reaction leading to reduced nitric oxide availability, increased markers of inflammation, and the chemical modification of lipoproteins [227,228].

Coenzyme Q10 (CoQ10), a lipid-soluble micronutrient that is endogenously synthesized in the body [229], appears to be a promising candidate for treating T2DM and its cardiovascular complications. It precisely targets oxidative stress, owing to its potent antioxidant activity and fundamental physiological role in mitochondrial bioenergetics [230]. CoQ10 has two primary functions: promoting ATP synthesis and serving as a potent antioxidant, making it one of the most active scavengers for ROS and providing protection to mitochondria membrane proteins, lipids, and DNA from oxidative damage. The reduced form of CoQ10, ubiquinol, serves as a potent antioxidant since it holds electrons loosely and can easily give up one or two electrons to neutralize ROS [229]. 

Given that CoQ10 is predominantly distributed within tissues with elevated energy requirements, its abundance is notable in specific dietary sources, particularly in animal hearts and livers and fatty fish, such as salmon, sardines, and herring, as well as in plant-based sources, like soybeans, spinach, and nuts, although in a lower concentration compared with meat and fish [231]. Unfortunately, the efficiency of absorption of orally administered CoQ10 is poor because of its insolubility in water, limited solubility in lipids, and relatively large molecular weight; endogenous synthesis is believed to be its main source [232].

However, when it comes to T2DM, there is more evidence that the supplementation of CoQ10 can support the treatment of type 2 diabetes and its complications. Many studies contradicting the theory of its beneficial effects were conducted in the 1990s, when ubiquinol, the reduced form of CoQ10, was not yet accessible as a supplement on the market. Instead, only ubiquinone, the oxidized form of CoQ10, was investigated [229]. What is known today is that depleted CoQ10 levels in the blood (serum and platelets) have been reported in type II diabetic patients [233] and the supplementation with 100 mg of ubiquinone twice daily (200 mg/day) or 100 mg of ubiquinol per day can significantly decrease HbA1C, as well as systolic and diastolic blood pressure for ubiquinone [234,235,236]. It aligns with another study’s results, indicating a significant negative correlation between CoQ10 and HbA1C concentrations [233]. However, data regarding its effect on fasting glucose, fasting insulin, and HOMA-IR are found to have low certainty of the evidence (GRADE Evidence Profile) because of inconsistency, indirectness, and publication bias [237]. This means that the positive impact of CoQ10 supplementation on the above factors cannot be confirmed with certainty, but these results should be taken into consideration, especially in the context of further research.

Alterations in mitochondrial function associated with the increased production of ROS have been attributed as significant factors in cardiovascular complications of T2DM, from endothelial dysfunction to heart failure. Several studies suggest that CoQ10 supplementation can counteract abnormal endothelial function by activating endothelial nitric oxide synthase (eNOS) and mitochondrial oxidative phosphorylation [238,239].

Promising outcomes of CoQ10 effectiveness have been presented in a recent Q-SYMBIO study. The potential benefits of ubiquinone supplementation (3 × 100 mg/day for two years) among patients with chronic heart failure (NYHA class III or IV) were assessed. Supplementation with CoQ10 reduced the risk of MACE (major adverse cardiovascular event) by 43%, the risk of cardiac-related death by 43%, and all-cause mortality by 42%. The results demonstrated that treatment with CoQ10, in addition to standard therapy for patients with moderate to severe HF, is safe, well tolerated, and associated with improvement in NYHA functional class [240].

Regarding diabetic polyneuropathy, a double-blind, placebo-controlled clinical trial was conducted. Patients were given 400 mg of ubiquinone a day for twelve weeks, resulting in significant improvement in clinical outcomes and nerve conduction parameters of diabetic polyneuropathy without adverse effects [241]. 

#### 5.4.2. Alpha-Lipoic Acid

Alpha-lipoic acid (ALA), also known as thioctic acid, was first isolated by L. J. Reed et al. from insoluble liver residues. The name of the compound was created based on the high solubility of ALA in fat and its acidity. Reed et al., based on the proved catalytic properties of lipoic acid in the aerobic decarboxylation of pyruvate, incipiently classified this compound to the family of B vitamins [242].

ALA is produced by both plants and animals, including humans. This compound, due to its single chiral center, exists in two forms: the R isomer and the S isomer. Both isomers occur in equal amounts in alpha-lipoic acid. It is worth mentioning that only the R isomer occurs naturally and the S isomer rises in the chemical reactions [243,244,245]. Alpha-lipoic acid is produced by humans from fatty acids and cysteine, but its endogenous production is not sufficient to ensure the proper functioning of cells, which means that this compound is delivered to the organism mainly through the diet [245,246]. Both animal and plant tissues contain alpha-lipoic acid in the form of the R isomer, and it occurs in a form bound to lysine residues, such as lipoyllysine. R-ALA occurs in the highest concentration in animal tissues, such as the kidneys, heart, and liver, while in plants, R-ALA is found in the highest concentration in spinach, broccoli, and tomatoes [244,246]. Alpha-lipoic acid available as a dietary supplement is a mixture of both racemic forms. Both ALA and its reduced form, dihydrolipoic acid (DHLA), have antioxidant properties and, if combined, may be referred to as the “universal antioxidant” [244]. It has been described that this universal antioxidant is instrumental in the regeneration of other antioxidants, including vitamin C, glutathione, vitamin E, and even dihydrolipoic acid, which neutralizes free radicals, does not degenerate, and is converted back to its oxidized form, alpha-lipoic acid [244,247,248].

Alpha-lipoic acid affects many crucial cellular pathways in the body. One of its actions is to influence the immune system by inhibiting the activation of nuclear factor kappa B. NF-κB is a protein complex that acts as a transcription factor, participating in the regulation of the immune response. NF-kB is found in most animal cells and is activated by various factors, such as cytokines, free radicals, viruses, bacteria, and stress [249,250]. It has been shown that dysregulation in the NF-κB pathway can be associated with disorders such as cancer, autoimmune diseases, and inflammatory and metabolic diseases [251,252,253]. 

Alpha-lipoic acid also serves a significant purpose as a regulator of gene transcription, modulated by peroxisome proliferator-activated receptors (PPARs). PPARs are substantially involved in cell differentiation and maturation processes, as well as metabolic processes involving carbohydrates, proteins, and lipids. The use of fibrates and glitazones in metabolic diseases, which also affect PPARs, confirms the fact that alpha-lipoic acid, through the aforementioned effect on PPARs, may also play a significant role in modifying the course of metabolic diseases, such as diabetes. 

Alpha-lipoic acid affects many pathways involved in the pathogenesis of diabetes mellitus. In diabetes, hyperglycemia causes increased production of ROS, which then causes cellular damage [249]. ROS negatively affects insulin signaling and contributes to the development of insulin resistance [254]. It has been shown that increased levels of ROS result in the formation of oxidized forms of LDL (ox-LDL), which are not properly recognized by LDL receptors, resulting in an increased concentration of ox-LDL in the blood. These forms are then phagocytosed by macrophages, which turn into foam cells and are deposited in the form of atherosclerotic plaques. It has been proven that ROS, by influencing endothelial cells, disturbs vasorelaxation, causes apoptosis of endothelial cells, increases the proliferation and migration of smooth muscles of blood vessels, and also promotes abnormal angiogenesis. These processes may be related to the micro- and macrovascular complications occurring in diabetes [249,255,256]. The antioxidant potential of ALA can prevent the adverse effects of ROS on cells and tissues.

It has been shown that ALA also affects the expression of 5’AMP-activated kinase (AMPK), which plays a significant role in the pathogenesis of diabetes. AMPK activation leads to reduced gluconeogenesis in the liver, increased glucose uptake, and fatty acid oxidation by skeletal muscles, which contributes to a reduction in blood glucose concentration and improved insulin sensitivity of cells [249,257]. AMPK also increases the amount of glucose-uptake transporters 4 on cell membranes in an insulin-independent mechanism, which also contributes to the hypoglycemic effect [248,249,257].

Studies on rats have proven that alpha-lipoic acid has an inhibitory effect on hypothalamic AMPK, causing a decrease in food intake and an increase in energy expenditure, contributing to a decrease in the body weight of the tested animals. This discovery opens the way for research on ALA as a potential drug against obesity, which itself is associated with an increased risk of type 2 diabetes [258,259].

It has been proven that alpha-lipoic acid taken orally has a bioavailability of approximately 29%, which is associated with a pronounced first-pass effect. It is worth mentioning that a meal reduces its bioavailability, so it is recommended that alpha-lipoic acid should be taken at least 30 min before the planned meal [260].

In many clinical trials involving humans, such as ALADIN, ORPIL, and SYDNEY, various doses of ALA were used, ranging from 200 to 1800 mg per day, but no specific dose has been established, and further research is necessary in this area. However, there were no contraindications to the use of alpha-lipoic acid, and side effects were limited only to hypersensitivity reactions [249].

#### 5.4.3. Omega-3 Fatty Acids

The main representatives of omega-3 acids are eicosapentaenoic acid (EPA) and docosahexaenoic acid (DHA) [261]. Reliable sources of omega-3 fatty acids are fish, such as salmon, tuna, mackerel, anchovies, and sardines [262]. The recommended daily intake ranges from 250 to 500 mg per day and depends on gender and age [263]. Omega-3 polyunsaturated fatty acids (n3-PUFAs), mediators of inflammation and adaptive immune responses, have anti-inflammatory and antioxidant properties [110,264]. Omega-3 fatty acids regulate immune responses by producing inflammatory cytokines. EPA influences increased glucose uptake in skeletal muscle cells and the regulation of insulin secretion pathways by pancreatic cells. There are studies that show that EPA supplementation reduces fasting plasma glucose, insulin, HbA1c, and HOMA-IR levels in patients with type 2 diabetes. It may also affect insulin regulation via PRAR-γ [265]. DHA can reduce blood glucose levels, but its impact on insulin regulation is related to its anti-inflammatory effect. Omega-3 acids, by secreting adipocytokines and affecting adipose tissue, can improve mitochondrial function and, as a result, improve insulin resistance. Some studies show that omega-3 fatty acids have a positive effect on insulin sensitivity in both diabetics and non-diabetics. One of them shows that increased consumption of omega-3 fatty acids was associated with a decrease in serum C-reactive protein levels. Supplementing with omega-3 fatty acids can improve insulin sensitivity, reduce inflammation, and may reduce the risk of developing diabetes [266].

## 6. Conclusions

The human body’s proper function depends not solely on its diet but also on the intake of nutrients and non-nutritive bioactive compounds. This applies not only to healthy individuals but especially to those with co-occurring chronic conditions, including type 2 diabetes. Unfortunately, the current food industry and the widespread use of highly processed food promote the development of nutritional deficiencies. A plethora of the aforementioned studies have confirmed the existence of these deficiencies. The foremost method of determining the demand for compounds essential for the adequate performance of the human body is the preliminary estimation of their concentration, which, in effect, is not possible to the full extent. However, widely available tests may be used in order to directly and indirectly assess the patient’s nutritional status. It is requisite to conduct a meticulous interview, especially taking into account eating habits, and determine the Body Mass Index (BMI). The tests that ought to be performed in every patient with diabetes include complete blood count with a smear (lymphopenia may be an indicator of malnutrition) and the concentration of total protein, albumin, folic acid, B12, ferritin, vitamin D3, calcium, uric acid, and lipid profile [218,219,267].

When a patient experiences inflammation and it is anticipated that the concentration of ferritin, an acute phase protein, may be elevated, it is recommended to perform the transferrin saturation test (TSAT), which requires the concentration of iron and unsaturated iron binding capacity. Research conducted by Pilar Vaquero M et al. showed that low TSAT levels are very prevalent in diabetes, primarily in women. If the TSAT is lower than 15% in men and 12% in women, iron deficiency may be diagnosed, and supplementation is necessary [268].

Research conducted by the author of this article on a group of patients with primary immunodeficiency revealed reduced hemoglobin concentration in 32%, total protein in 19%, albumin in 17%, vitamin D3 in 52% (despite recommended supplementation), vitamin B12 in 6.5%, folic acid in 34%, and ferritin in 26% of patients. It is important to note that the study was performed on a group of patients who regularly attend medical appointments and undergo periodical examinations [269]. Diabetes is a secondary immune deficiency, and patients who have been diagnosed with it should also be carefully examined for nutritional deficiencies. The research conducted in patients with recurrent infections in the Immunology Clinic (including type 2 diabetes) and the implementation of periodic vitamin and mineral supplementation (for 2 months, then every 2–3 months for a month), the additional regular supplementation of vitamin D3, calcium, and omega-3 fatty acids significantly improved the functioning of patients, both clinically and in the laboratory (data not yet published).

Supervision of consumption behavior and assessment of diabetic patients for nutritional deficiencies should be instrumental in the care of this group of patients. Appropriate nutrition affects the effective activity of the immune system, ensuring homeostasis of the entire body, diminishes the development of complications, reduces the risk of other chronic diseases, and thus improves the length and quality of patients’ lives.

## Figures and Tables

**Table 1:** Novel antidiabetic drugs.

Antidiabetic Drug	Mechanism of Action	Function
Tirzepatide	Dual agonist of GIP and GLP-1 receptors	Reduces HbA1c Reduces body weight Improved cardiometabolic risk factors
Pioglitazone	PPARγ agonist	Decreased risk of myocardial infarctions, ischemic strokes, and major adverse cardiovascular events
DA-1241	GPR119 receptor agonist	Improves glucose-dependent insulin release by the stimulation of GLP-1 secretion Reduces hepatic gluconeogenesis
Vaspin	Kallikrein 7 inhibitor	Improves insulin sensitivity and glucose tolerance and inhibits insulin degradation Modulates adipocyte differentiation and reduces inflammatory adipokines
Metrnl	PPARγ receptor agonist	Increases lipid metabolism Improves insulin resistance Reduces inflammation
Fetuin-A	The extracellular portion of the transmembrane β-subunit of the insulin receptor (InsR)	Modulates insulin receptor signaling and influences glucose homeostasis Correlated with the incidence of t2dm and obesity-related complications Higher levels associated with insulin resistance

## Data Availability

No new data were created or analyzed in this study. Data sharing is not applicable to this article.
